# Early Thoracotomy and Decortication in Pleural Empyema

**DOI:** 10.7759/cureus.93879

**Published:** 2025-10-05

**Authors:** Arvind Kohli, Vishal V Bhende, Amit Chaudhary, Viral B Patel, Mathangi Krishnakumar, Swati Roy

**Affiliations:** 1 Cardiothoracic and Vascular Surgery, Swami Vivekananda Medical Mission Charitable Hospital, Jammu, IND; 2 Pediatric Cardiac Surgery, Bhanubhai and Madhuben Patel Cardiac Centre, Shree Krishna Hospital, Bhaikaka University, Anand, IND; 3 Pediatric Cardiac Surgery, Sri Sathya Sai Sanjeevani Centre for Child Heart Care and Training in Pediatric Cardiac Skills, Navi Mumbai, IND; 4 Vascular Surgery, King George's Medical University, Lucknow, IND; 5 Radiodiagnosis, Pramukhswami Medical College, Shree Krishna Hospital, Bhaikaka University, Anand, IND; 6 Anesthesiology, St. John's Medical College Hospital, Bengaluru, IND; 7 Epidemiology and Public Health, Amrita Patel Centre for Public Health, Bhaikaka University, Anand, IND

**Keywords:** decortication, open thoracotomy, pleural empyema, pleural infection, video-assisted thoracoscopic surgical decortication

## Abstract

Background and aim

Pleural empyema remains a serious complication of pulmonary infections, with high morbidity and mortality if not managed effectively. While antibiotics and drainage are sufficient in the early stages, chronic empyema often requires surgical intervention. This study evaluates the outcomes of early thoracotomy and decortication for pleural empyema at a tertiary cardiac center in India.

Methods

A retrospective observational study was conducted on nine patients who underwent early thoracotomy and decortication between 2018 and 2024. Demographics, radiological findings, etiology, complications, survival, and follow-up outcomes were analyzed. Survival and event-free survival were assessed using the Kaplan-Meier method.

Results

Six patients were female (66.7%), and three were male (33.3%). Four patients underwent right-sided, four left-sided, and one bilateral decortication. Six patients (66.7%) had moderate pleural fluid, three (33.3%) had large fluid collections, and all nine (100%) had pleural thickening ≥2 cm. Etiologies included non-tuberculosis (TB; n = 7; 77.8%) and TB (n = 2; 22.2%). Postoperative complications occurred in three patients (33.3%), including prolonged air leak (n = 2) and superficial wound infection (n = 1). One patient (11.1%) died from nosocomial pneumonia, acute respiratory distress syndrome, and septic shock. Kaplan-Meier estimated survival probability was 88.9% at 30 days and remained stable at 12 months. Event-free survival at 12 months was 66.7%. The median hospital stay was 12 days (range, 8-21). All survivors demonstrated satisfactory lung re-expansion with no recurrence during three to 12 months of follow-up.

Conclusions

Early thoracotomy and decortication is a safe and effective treatment for advanced-stage pleural empyema, yielding favorable survival and functional outcomes. Despite advances in minimally invasive surgery, thoracotomy remains indispensable for selected patients, particularly in TB-endemic regions.

## Introduction

Pleural empyema, defined as the accumulation of pus in the pleural cavity, has been recognized since Hippocratic times [1,2]. Despite advances in antimicrobials and interventional techniques, empyema remains a major clinical problem with significant morbidity and mortality [3,4]. Its incidence has increased globally due to antibiotic resistance, delayed diagnosis, and comorbidities [5].

The disease typically progresses through three stages: exudative, fibrinopurulent, and organizing fibrothorax [6,7]. While early disease may respond to antibiotics and chest drainage, advanced stages often require surgical intervention [8].

Video-assisted thoracoscopic surgery (VATS) is increasingly preferred for fibrinopurulent empyema [9,10]. However, in organizing empyema with a thick pleural peel and lung entrapment, VATS may be insufficient, and open thoracotomy with decortication remains the gold standard [11-13]. Decortication restores lung expansion, improves pulmonary function, and reduces recurrence [14,15].

In TB-endemic countries such as India, tuberculous empyema adds further complexity, with delayed presentations frequently requiring thoracotomy [16-19]. Indian pediatric data, including findings from Gandotra et al., demonstrate that early surgical referral improves outcomes compared with delayed intervention [18].

A recent bibliometric analysis by Bhende et al. highlighted a surge in global research on the surgical management of empyema, with thoracotomy and decortication remaining highly relevant despite advances in VATS [20]. This study reports a single-center experience of early thoracotomy and decortication for empyema, focusing on survival, complications, and functional outcomes in the Indian TB-endemic setting.

## Materials and methods

This study was designed as a retrospective observational analysis of patients who underwent early thoracotomy and decortication for pleural empyema between 2018 and 2024 at the Bhanubhai and Madhuben Patel Cardiac Centre, Karamsad, Gujarat, India. Ethical clearance was obtained from the Institutional Ethics Committee-2 of Bhaikaka University, Anand, under approval number IEC/BU/2024/Cr.31/276/2025, dated September 2, 2025.

Clinical and operative data were extracted from a computerized cardiovascular database (FTP 192.168.0.5). Information on patient demographics, laterality of surgery, pleural fluid level, pleural thickening, etiology, postoperative complications, and clinical outcomes was collected and analyzed.

Patients were included if they had a confirmed diagnosis of pleural empyema requiring surgical decortication, were younger than 12 years, and had failed to respond adequately to conservative management with antibiotics and drainage. Patients with malignant pleural effusions or those who were severely immunocompromised and considered poor surgical candidates were excluded.

Data were analyzed using descriptive statistical methods. Categorical variables were expressed as absolute frequencies and percentages, while continuous variables were reported as medians with ranges. Survival and event-free survival were assessed using Kaplan-Meier-style reporting, with probabilities calculated at 30 days and 12 months after surgery.

## Results

A total of nine patients were included in the study, consisting of six females (66.7%) and three males (33.3%) (Table 1).

**Table 1:** Patient characteristics in our study Mild: occupying <25% of hemithorax volume; moderate: occupying 25-50% of hemithorax volume; high: occupying >50% of hemithorax volume +, present; −, absent TB, tuberculosis

Sr. no.	Patient	Age	Sex	Laterality (decortication)	Pleural fluid level			Pleural thickening		Etiology		Outcome
					Mild	Moderate	High	<2 cm	≥2 cm	TB	Non-TB	
1	1	6	M	Right	+	-	-	-	+	-	+	Expired
				Left	-	+			+			
2	2	4	F	Left	-	-	+	-	+	-	+	Discharged
3	3	10	F	Right	-	+	-	-	+	+	-	Discharged
4	4	9	F	Right	-	-	+	-	+	+	-	Discharged
5	5	8	F	Left	-	+	-	-	+	-	+	Discharged
6	6	2	F	Right	-	+	-	-	+	-	+	Discharged
7	7	5	F	Left	-	+	-	-	+	-	+	Discharged
8	8	9	M	Left	-	-	+	-	+	-	+	Discharged
9	9	11	M	Right	-	+	-	-	+	-	+	Discharged

The cohort had a median age of eight years (IQR 4.5-9.5). Surgical laterality was evenly distributed, with four patients (44.4%) undergoing right-sided decortication, four (44.4%) undergoing left-sided decortication, and one (11.1%) requiring bilateral intervention. Radiological and intraoperative assessments showed moderate pleural fluid in six patients (66.7%) and high pleural fluid in three (33.3%). All nine patients (100%) demonstrated pleural thickening ≥2 cm, consistent with advanced empyema. The underlying etiology was non-tuberculous in seven patients (77.8%) and tuberculous in two (22.2%) (Table 2).

**Table 2:** Patient characteristics and outcomes TB, tuberculosis

Parameter	Findings (n = 9)
Sex	Female: 6 / Male: 3
Laterality	Right: 4 / Left: 4 / Bilateral: 1
Pleural fluid	Moderate: 6 / High: 3
Pleural thickening	≥2 cm: 9
Etiology	TB: 2 / Non-TB: 7
Complications	Three patients (prolonged air leak, superficial wound infection)
30-day mortality	One patient
12-month survival probability	88.90%
Event-free survival (12 months)	66.70%
Median hospital stay	12 days (range 8-21)

**Figure 1:**
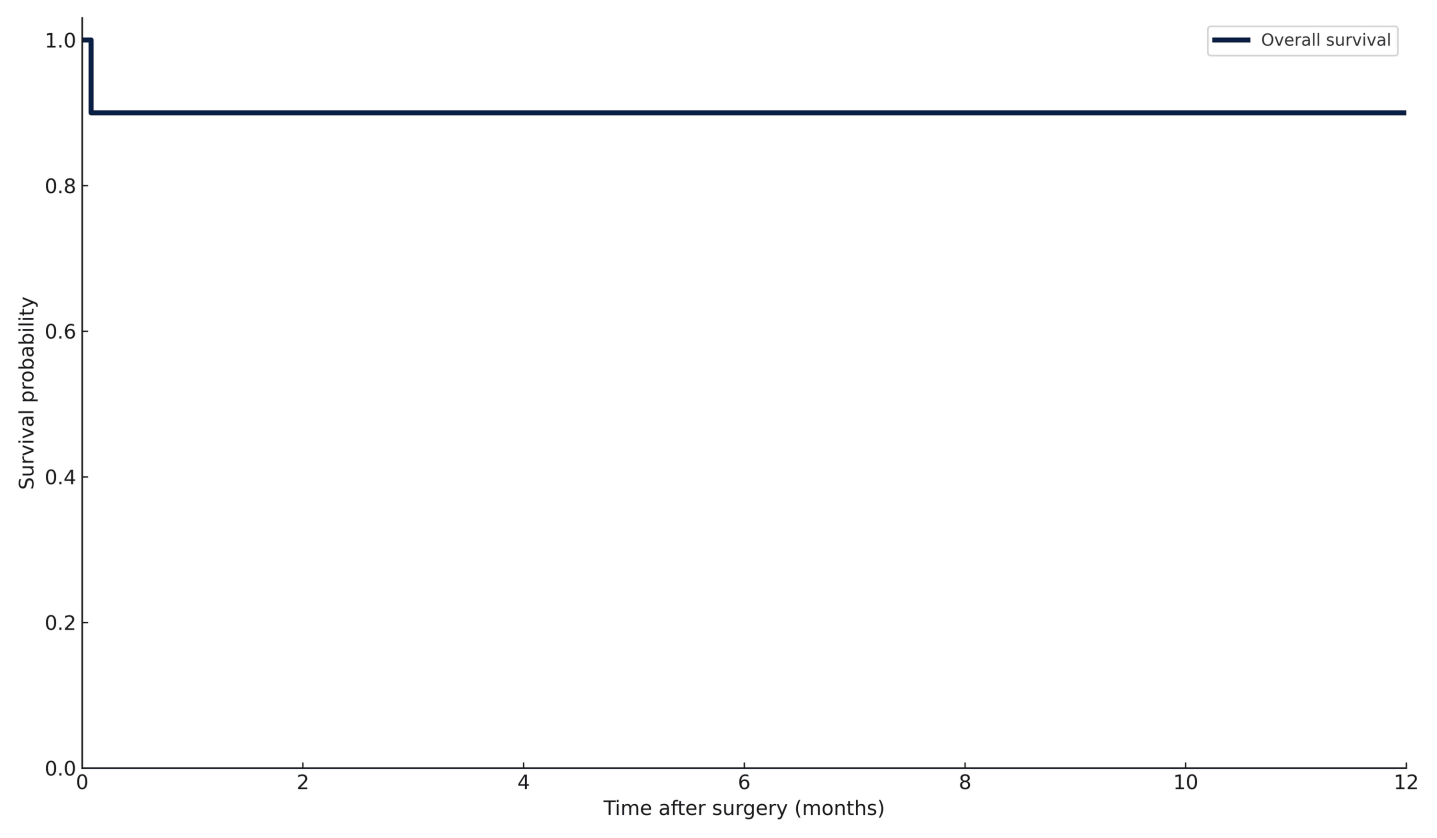
Kaplan-Meier overall survival curve

Postoperative complications occurred in three (33.3%) patients, including prolonged air leak in 2 (22.2%) and superficial wound infection in 1 (11.1%); all were managed conservatively. One patient (11.1%) died within five days of surgery due to acute respiratory distress syndrome and septic shock, corresponding to a 30-day mortality rate of 11.1%. The Kaplan-Meier survival probability was 88.9% at 30 days and remained unchanged at 12 months (Figure 1).

**Figure 2:**
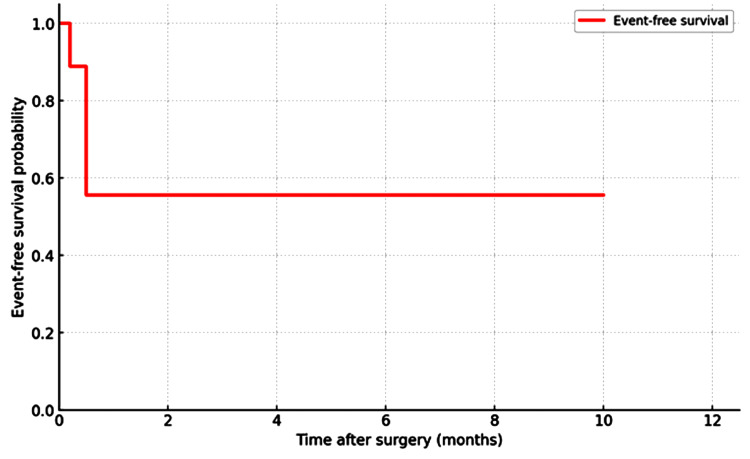
Kaplan-Meier event-free survival curve

Event-free survival, defined as freedom from death or major complications, was 66.7% at 12 months (Figure 2).

The median postoperative hospital stay was 12 days (range, 8-21 days). At follow-up (three to 12 months), all surviving patients demonstrated radiological evidence of complete lung re-expansion and had returned to baseline functional status, with no recurrence of empyema.

**Figure 3:**
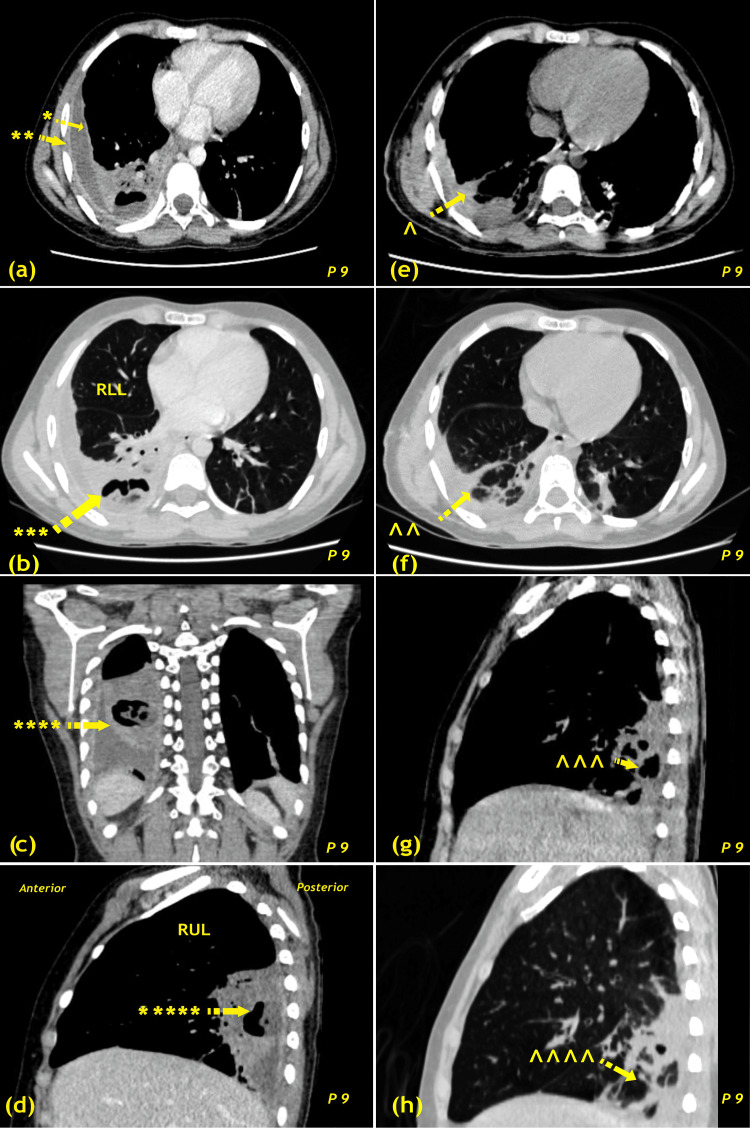
Pre- and postoperative CT scans Preoperative: (a) CECT thorax, axial mediastinal window shows right-sided loculated mild pleural effusion (*) with thickened enhancing visceral and parietal pleura (**); (b) CECT thorax, axial lung window shows dense consolidation with internal air bronchogram and cavitation with air-fluid levels in the right lower lobe, suggestive of developing abscess (***); (c) CECT thorax, coronal mediastinal window shows right-sided loculated mild pleural effusion with thickened enhancing pleura and developing abscess (****); (d) CECT thorax, sagittal mediastinal window shows similar findings (*****). Postoperative: (e) HRCT thorax, axial mediastinal window shows significant reduction in pleural effusion with resolving consolidation (^); (f) HRCT thorax, axial lung window shows resolving consolidation (^^); (g) HRCT thorax, sagittal mediastinal window shows decrease in cavitation size (^^^); (h) HRCT thorax, sagittal lung window image (^^^^). CECT, contrast-enhanced CT; HRCT, high-resolution CT; P, patient; RLL, right lower lobe; RUL, right upper lobe; s/o, suggestive of Image credit: Viral B. Patel

**Figure 4:**
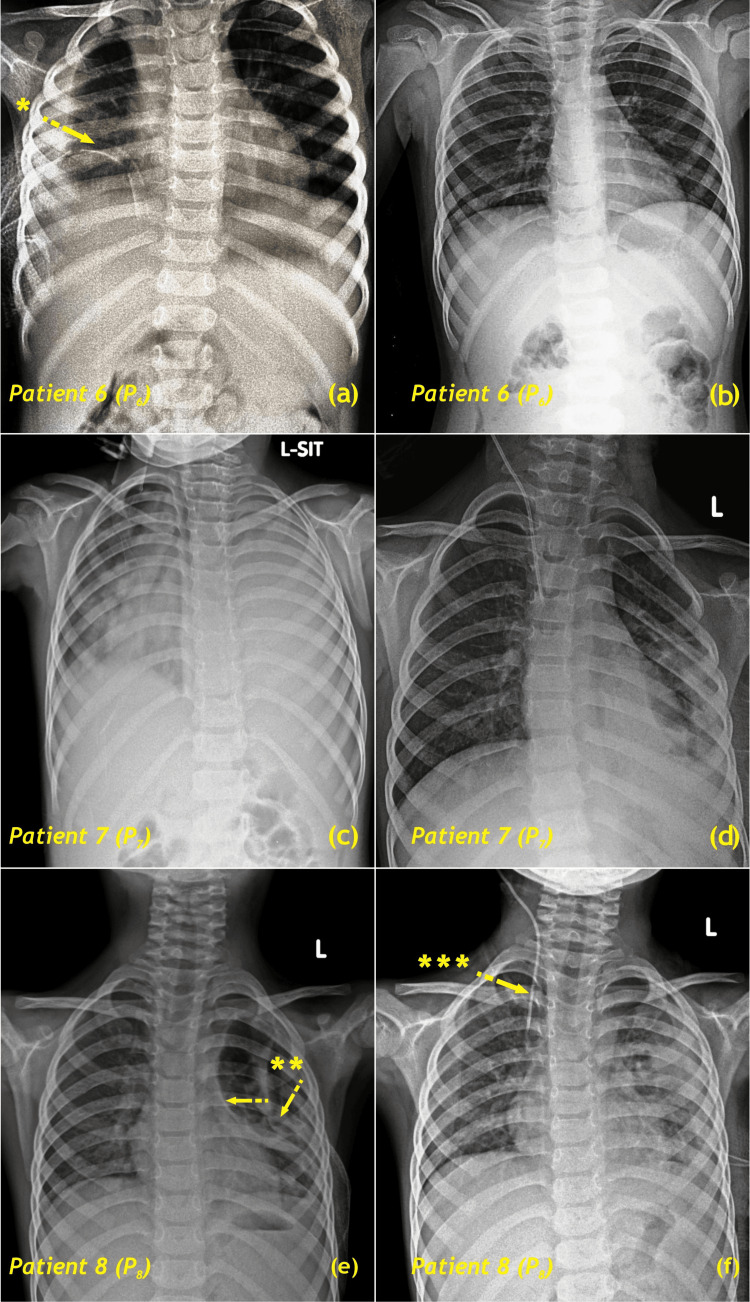
Chest radiographs pre- and postoperative Right decortication (P6), left decortication (P7, P8). (a, c, e) Preoperative; (b, d, f) postoperative. *, right intercostal drain; **, loculated empyema; ***, central venous pressure line Image credit: Vishal V. Bhende

Representative imaging is shown in Figure 3 (pre- and postoperative CT scans from a typical advanced empyema case with dense pleural cortex and trapped lung) and Figure 4 (pre- and postoperative chest radiographs from three patients who underwent right (P6) and left (P7, P8) decortication). These images illustrate multiloculated collections, thickened visceral and parietal pleura, and restoration of lung expansion following decortication.

## Discussion

Our findings indicate that early thoracotomy and decortication yield favorable outcomes in patients with pleural empyema, with a 12-month overall survival of 88.9% and an event-free survival of 66.7%. These results are consistent with prior studies emphasizing the importance of early surgical referral in reducing morbidity and facilitating restoration of lung function [11-14]. In our series, all survivors demonstrated radiological lung re-expansion and regained their preoperative functional capacity.

Radiological evaluation remains fundamental for operative planning. Contrast-enhanced CT is particularly reliable for identifying pleural thickening, loculations, and trapped lung [9,16,17]. In the present cohort, all patients exhibited pleural thickening ≥2 cm, a radiological marker consistent with advanced empyema and a clear indication for surgical decortication.

The choice between VATS and thoracotomy remains debated. VATS is widely accepted as useful in the fibrinopurulent stage [9,10], yet in the setting of organizing empyema, it may be technically inadequate [12,13]. Since all patients in our series presented with advanced disease, thoracotomy was the logical approach. This is supported by systematic reviews demonstrating that thoracotomy allows for more complete decortication in chronic cases [13].

The observed mortality of 11.1% falls within the reported range of 5-15% for advanced empyema [18]. Postoperative morbidity was limited to 33.3% of patients and comprised prolonged air leaks and a single wound infection, all of which were managed conservatively.

Tuberculous empyema remains a particular challenge in India due to late presentation and dense pleural peel. Both tuberculous patients in this study benefited from decortication, reflecting the continued relevance of surgery in such cases. In our cohort of nine patients, elevated lactate dehydrogenase levels and positive pleural fluid cultures indicated prolonged disease duration.

Previous studies have demonstrated pleural cortex thickness ≥2 cm and thoracic cage constriction as markers of chronicity. Chest CT has proven invaluable for visualizing the pleural cortex, identifying loculations, and accurately measuring empyema size. While there are no definitive indicators for chronic empyema [17], our series found that a thickened pleural cortex, thoracic cage constriction, and a disease course exceeding 15 days suggest chronic disease. Additionally, chest CT helps determine the most suitable surgical approach, such as limited or posterolateral thoracotomy. Routine preoperative chest CT scans have been performed for all empyema patients since 2018.

Our study also revealed that patients in the surgical treatment group demonstrated better performance status and fewer comorbidities. Surgical decortication, involving fibrous tissue excision and clearance of pus and debris [12], is particularly challenging in patients with poor health. Nevertheless, it is well established that decortication does not prolong hospital stay.

Open thoracotomy and decortication demonstrated high efficacy in our study, with low morbidity and mortality rates. Patients achieved excellent functional outcomes and subsequently resumed their pre-surgery activities. Open thoracotomy and decortication remain standard treatments for chronic empyema; however, direct comparisons with video thoracoscopy in prospective, randomized studies are lacking. Such research is essential to optimize patient selection for each procedure.

Gandotra et al. previously showed that early surgical intervention in pediatric empyema significantly improves outcomes compared with delayed intervention [18,19].

At the global level, bibliometric analysis by Bhende et al. demonstrated an increasing trend in publications on surgical management of empyema, with thoracotomy and decortication continuing to maintain relevance despite advances in minimally invasive techniques [20-22]. The present study reinforces these findings, particularly in the context of TB-endemic regions where advanced empyema is common and thoracotomy remains indispensable.

Limitations

This study has several limitations. First, it was a retrospective, single-center analysis with a small sample size of nine patients, which limits statistical power and generalizability. The absence of a control group undergoing alternative modalities, such as VATS or intrapleural fibrinolysis, precludes direct comparison of outcomes between open and minimally invasive techniques. In addition, pulmonary function tests and long-term quality-of-life assessments were not systematically performed, making it difficult to objectively quantify functional recovery beyond radiological expansion and clinical follow-up. Another limitation is the potential for selection bias, as patients referred for thoracotomy were already deemed unsuitable for conservative management or VATS, which may have skewed the outcomes toward more advanced disease. Finally, follow-up was limited to three to 12 months, preventing assessment of late recurrence or long-term functional decline. Larger, prospective, multicenter studies with standardized functional evaluation are needed to validate these findings and refine patient selection criteria.

## Conclusions

Early thoracotomy and decortication provided a reliable source control and lung re-expansion in this cohort of advanced empyema, radiologically characterized by thick pleural cortex, yielding favorable short-term survival and functional recovery, with a 30-day and 12-month overall survival of 88.9% and an event-free survival of 66.7% at 12 months. Perioperative morbidity was acceptable and managed conservatively. These outcomes support timely surgical referral when medical therapy and drainage are unlikely to achieve re-expansion or when imaging suggests a trapped lung, underscoring the ongoing importance of open surgery for complex disease, including tuberculous empyema in TB-endemic settings. Rather than positioning thoracotomy against minimally invasive approaches, our experience suggests a complementary strategy in which VATS is employed when feasible, with open decortication reserved for dense pleural peel, multiloculation, or failure of less invasive management.
